# Fluorescence-aided identification technique (FIT) in retainer removal: Effects on enamel loss, composite residue, and time

**DOI:** 10.1007/s00056-025-00606-w

**Published:** 2025-08-13

**Authors:** Henriette Ida Ulrike Stasche, Inga Vanessa Bordihn, Ellen Schulz-Kornas, Rainer Haak, Tobias Meissner, Julian Petersen, Cita Nottmeier, Till Koehne

**Affiliations:** 1 Department of Orthodontics, Leipzig University, Leipzig, Germany; 2 Department of Cariology, Endodontology and Periodontology, Leipzig University, Leipzig, Germany

**Keywords:** Optical coherence tomography, Orthodontic fixed retainer, Composite residue, Enamel loss, Bovine teeth, Optische Kohärenztomographie, Festsitzender kieferorthopädischer Retainer, Kompositrückstände, Zahnschmelzverlust, Rinderzähne

## Abstract

**Purpose:**

The aim of this study was to investigate the difference in enamel loss, composite residue, and time during removal of fixed orthodontic retainers using ultraviolet (UV) light compared to standard white light.

**Methods:**

A nickel–chromium retainer was bonded to 4 bovine teeth using a fluorescence-modified composite (Aligner Flow Composite, Voco, Cuxhaven, Germany); 20 models were created. In a split-mouth design, 2 teeth per retainer were removed using standard white light (non-FIT) and 2 teeth using the fluorescence-aided identification technique (FIT) with UV light. Removal time was recorded for each tooth. Intraoral and optical coherence tomography (OCT) scans were taken of the tooth before bonding (T0) and after retainer removal (T1). The intraoral scans were analyzed using the software Avizo to quantify enamel loss (T0–T1) and composite residue (T1–T0). The OCT scans were evaluated using a dichotomous scoring system for enamel loss and a four-point scoring system for composite residue.

**Results:**

There was no significant difference in removal time for the two groups. The overlay of intraoral scans showed significantly less enamel loss in area (39.35 ± 39.98 mm^2^ vs. 65.44 ± 58.11 mm^2^, *p* = 0.0235) and volume (3.41 ± 6.15 mm^3^ vs. 8.86 ± 11.65 mm^3^, *p* = 0.0116), as well as less composite residue in area (8.22 ± 16.04 mm^2^ vs. 30.87 ± 49.65 mm^2^, *p* = 0.0083) and volume (0.41 ± 0.87 mm^3^ vs. 2.34 ± 3.94 mm^3^, *p* = 0.0037) in the FIT group compared to standard white light. While OCT scans showed no significant difference in enamel loss, it proved that the amount of composite residue was significantly less in the FIT group compared to the standard light controls (5.88 ± 10.54 vs. 22.66 *p* = 0.0072).

**Conclusion:**

Although the FIT method does not reduce the removal time of orthodontic fixed retainers, it has been shown to be beneficial in preserving enamel and leaving less composite residue.

## Abstract (de)

**Zielsetzung:**

Ziel der Studie war es, den Unterschied zwischen Schmelzverlust, Kompositrückständen und Zeitaufwand bei der Entfernung von festsitzenden kieferorthopädischen Retainern mit ultraviolettem (UV) Licht im Vergleich zu normalem Weißlicht zu untersuchen.

**Methoden:**

Ein Nickel-Chrom-Retainer wurde mit einem fluoreszenzmodifizierten Komposit (Aligner Flow Composite, Voco, Cuxhaven, Deutschland) auf 4 Rinderzähne geklebt, 20 Modelle wurden erstellt. In einem Split-Mouth-Design wurden 2 Zähne pro Retainer mit normalem Weißlicht (non-FIT) und 2 Zähne mit der fluoreszenzgestützten Identifikationstechnik (FIT) mit UV-Licht entfernt. Die Entfernungszeit wurde für jeden Zahn aufgezeichnet. Von den Zähnen wurden intraorale und OCT(optische Kohärenztomographie)-Scans vor der Verklebung (T0) und nach der Entfernung des Retainers (T1) angefertigt. Die intraoralen Scans wurden mit der Software Avizo analysiert, um Schmelzverlust (T0-T1) und Kompositrückstände (T1-T0) zu quantifizieren. Die OCT-Scans wurden anhand eines dichotomen Bewertungssystems für den Zahnschmelzverlust und eines Vier-Punkte-Bewertungssystems für Kompositrückstände ausgewertet.

**Ergebnisse:**

Es gab keinen signifikanten Unterschied in der Entfernungszeit für beide Gruppen. Die Überlagerung der intraoralen Scans zeigte signifikant weniger Schmelzverlust in Fläche (39,35 ± 39,98 mm2 vs. 65,44 ± 58,11 mm2, *p* = 0,0235) und Volumen (3,41 ± 6,15 mm3 vs. 8,86 ± 11,65 mm3, *p* = 0. 0116) sowie weniger Kompositrückstände in Fläche (8,22 ± 16,04 mm2 vs. 30,87 ± 49,65 mm2,* p* = 0,0083) und Volumen (0,41 ± 0,87 mm3 vs. 2,34 ± 3,94 mm3, *p* = 0,0037) in der FIT-Gruppe im Vergleich zum Standard-Weißlicht. Während die OCT-Scans keinen signifikanten Unterschied im Schmelzverlust zeigten, konnte nachgewiesen werden, dass die Menge an Kompositrückständen in der FIT-Gruppe im Vergleich zu den Kontrollen mit Standardlicht signifikant geringer war (5,88 ± 10,54 vs. 22,66 *p* = 0,0072).

**Schlussfolgerung:**

Auch wenn die FIT-Methode die Entfernungszeit von festsitzenden kieferorthopädischen Retainern nicht verkürzt, hat sie sich als vorteilhaft für die Erhaltung des Zahnschmelzes erwiesen und hinterlässt weniger Kompositrückstände.

## Introduction

Fixed retainers were first introduced into dentistry in 1973 [17] and have since proven to be a good retention device for completed orthodontic treatment when used with the right indication [26, 41] and patient clientele. However, since the anterior teeth are particularly sensitive to movement, the fixed retainer can also cause unwanted movements of the incisors [18, 19, 25]. This, together with the failure of the composite bond [18], is often the reason why the fixed retainer needs to be removed in the first 3–6 months [18, 24, 41]. The average survival rate of an oral retainer is only between 68.4% [24] and 63% [24, 43], when observed over a maximum period of time of 41.7 months [24]. Therefore, removal of the retainers is a common procedure in orthodontic practice. However, this procedure presents new challenges. Incomplete removal of the composite used to bond the retainer to the teeth can compromise the bond strength for new retainers [3, 34], increasing the risk of failure, and can lead to higher plaque build-up rates [4, 36] if a new retainer is not placed. Multiple retainer removals also put the tooth substance at risk [7], as there is currently no technique to remove composite from the tooth without damaging the enamel [11, 15, 16, 40, 47] and leaving it in a sound condition. Therefore, the ultimate goal would be a technique that removes all of the composite and adhesive while preserving the tooth substance.

The fluorescence-aided identification technique (FIT), which uses the difference in light emission from tooth and composite to distinguish between the two [28, 29, 45], has been used in forensics since 1985 [8] and has recently been shown to be beneficial in the removal of composite fillings [6, 9, 20], brackets [13, 31, 42], trauma splints [10, 47], and even veneers [49]. It reduces removal time, enamel loss, and composite residue. Achieving a sensitivity of up to 84–97% and a specificity of up to 94–100% [23, 29].

Oral and buccal enamel differ at the microscopic level [5, 48] and working on the oral side has its own challenges that every dentist and orthodontist is familiar with. FIT could therefore be useful in overcoming the problems associated with frequent retainer removal. The aim of this study was to investigate the difference in enamel loss, composite, and adhesive residue and time during removal of fixed orthodontic retainers using UV light compared to standard white light.

The null hypothesis was that there would be no difference for the removal of the composite between applying FIT or standard white light with respect to enamel loss, as well as to composite and adhesive residue or time.

## Materials and methods

### Model preparation and digitalization

The bovine teeth used in this experiment were obtained from a butcher shop and are a byproduct of the meat industry. No animal was harmed with the sole purpose of conducting this study. The ethics committee from the medical faculty of the university of Leipzig refrains from demanding an ethics vote for in vitro studies in such cases. The quality control of the butcher shop, from which the teeth were received, is carried out by the Dezernat 3, Fachdienst Veterinärwesen, Potsdam Mittelmark (Control number veterinarian: 69001).

**Fig. 1:**
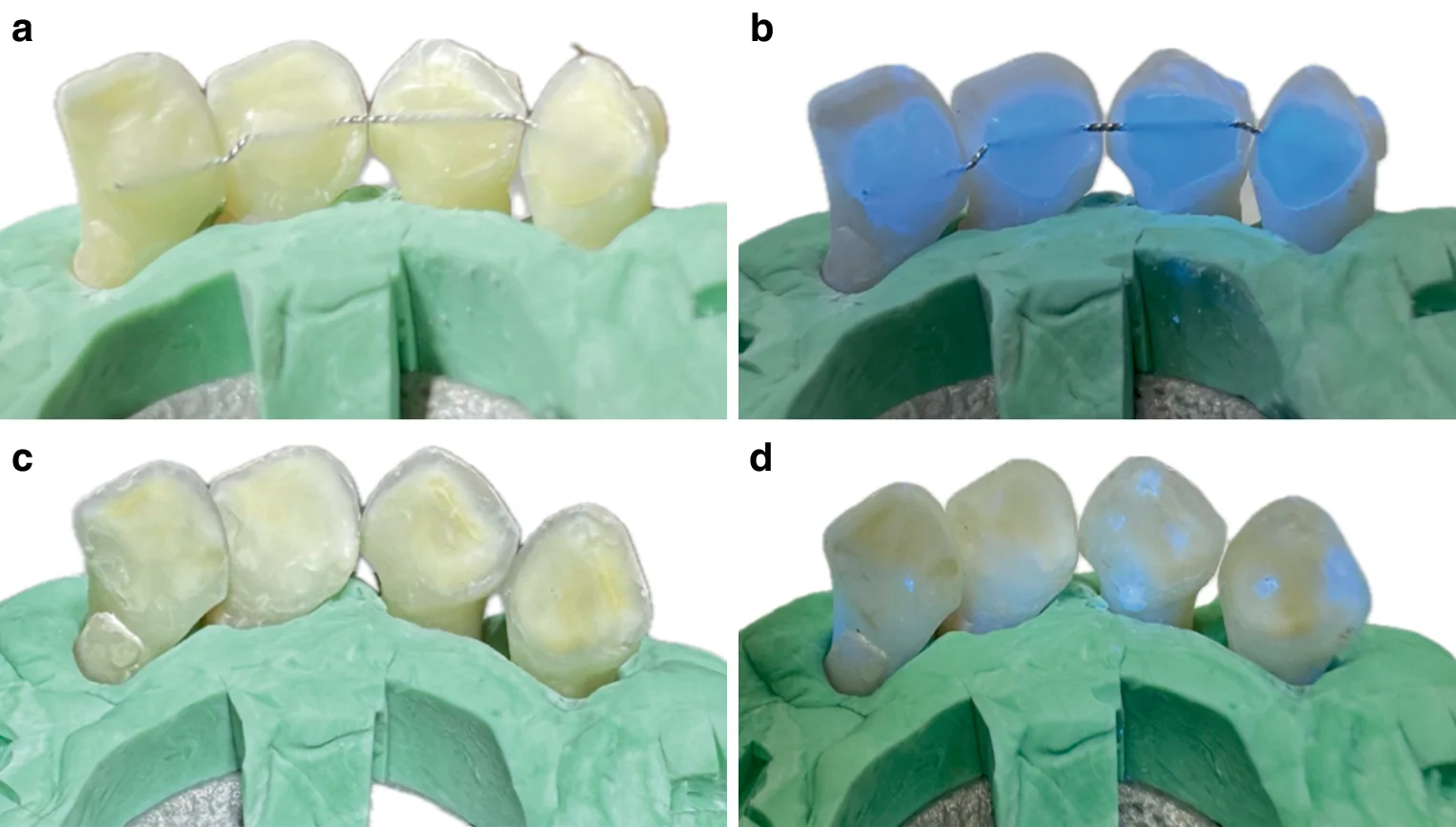
Trial setup. **a** Retainer attached to bovine incisors under standard light. **b** Retainer attached to bovine incisors under ultraviolet (UV) light. **c** Bovine teeth after retainer removal under standard light. **d** Bovine teeth after retainer removal under UV light showing the composite residue. Composite was removed from teeth in the third quadrant using the fluorescence-aided identification technique (FIT) and from teeth in the fourth quadrant under standard light Versuchsaufbau. **a** Retainer an Rinderschneidezähnen unter Standardlicht. **b** Retainer an Rinderschneidezähnen unter ultraviolettem (UV) Licht. **c** Rinderzähne nach Entfernung des Retainers unter Standardlicht. **d** Rinderzähne nach Entfernung des Retainers unter UV-Licht mit Kompositrückständen. Das Komposit wurde von den Zähnen im dritten Quadranten mit Hilfe der fluoreszenzgestützten Identifizierungstechnik (FIT) und von den Zähnen im vierten Quadranten unter Standardlicht entfernt

Bovine permanent first and second incisors were extracted from the lower jaw. Following a thorough cleaning and disinfection process, the teeth were stored in a 2% ammoniochloride solution in a standardized manner. Teeth that appeared to be cracked, demineralized, or otherwise unsuitable for the trial were excluded from the study. The sample size was calculated with G*Power 3.1.9.7 beforehand using the standard formula for two proportions [14]. Values needed were extracted from previous studies also investigating FIT [10, 13, 38, 42]. Each tooth was then three-dimensionally (3D) scanned (intraoral surface scanner, Trios 3, 3shape, Copenhagen, Denmark) and an optical coherence tomography (OCT; spectral-domain optical coherence tomography, Telesto II SP‑2, center wavelength 1310 nm ± 120 nm, sensitivity ≤ 106 dB, axial/lateral resolution < 5.5/20 μm [air], field of view ≤ 8 mm × 6 mm × 3.52 mm [pixel size 10 µm × 20 µm × 3.44 µm], imaging speed 28 kHz, A‑scan average 1, software ThorImage version 4.4; Thorlabs GmbH, Dachau, Germany) scan was recorded (T0). The scanning device was calibrated before starting the scanning procedure. A mold for precise positioning pre- and posttreatment was molded for each tooth. The OCT scans were then used to exclude teeth that did not meet the inclusion criteria of an enamel thickness over 0.15 mm for more than 50% of the tooth. Following this, the teeth were sorted into dental arches of four teeth per arch and fixed into a model (A‑S metal basis, Frasaco, Tettnang, Germany) using putty (Eurosil max lab Z putty, Henry Schein, Melville, NY, USA; Fig. 1). A total of 20 models were created. Following this, the finished dental arches (Fig. 1c) were scanned again, and a lingual fixed retainer was constructed using the software Onyxceph (version 3.2.223 (608); Image Instruments GmbH, Chemnitz, Germany). The nickel–chromium retainer (dentaflex straight wire 0.38 mm, Dentarium, Ispringen, Germany) was then bent and attached to the teeth using 33% phosphoric acid (Kaniedenta, Herfordt, Germany) to condition the surface, followed by a bonding adhesive (Transbond XT, 3M, Saint Paul, MN, USA) and a fluorescent tooth-colored composite (Aligner Flow Composite A2, VOCO, Cuxhaven, Germany) for bonding (Fig. 1a). The composite was light cured for a duration of 20 s, in accordance with the manufacturer’s guidelines.

### Retainer debonding

Two right-handed operators participated in the removal process. They were instructed to remove the retainer and the composite used for bonding until no residue could be identified visually. The 20 models were divided on each participant taking variation in size and form into consideration. The removal process was executed in a training model (PK‑2; Frasaco, Tettnang, Germany), which was designed to mimic clinical conditions. The retainer was removed using a split mouth method, with two teeth per arch being worked on using standard white light and being removed, before FIT was used on the two remaining teeth. The FIT procedure was executed using a handheld UV light (VOCO UV lamp, wavelength: 365 nm ± 5 nm; VOCO GmbH, Cuxhaven, Germany). The position of the teeth processed with FIT was alternated between each dental arch. The retainer was debonded using a tungsten carbide instrument (Komet Dental, Lemgo, Germany) at 40,000 rpm with water cooling and the teeth were polished using a brownie and greenie (ShoFu, Ratingen, Germany) at 20,000 rpm. A new drill tool was used for every eight teeth, and a new set of polishing instruments was used every four teeth. The presence of composite and adhesive remnants was detected using a dental probe and mirror, no magnification of any kind was allowed to help with the process. The time taken per tooth for retainer removal and polishing was recorded in seconds.

### Surface and tooth material analyses

**Fig. 2:**
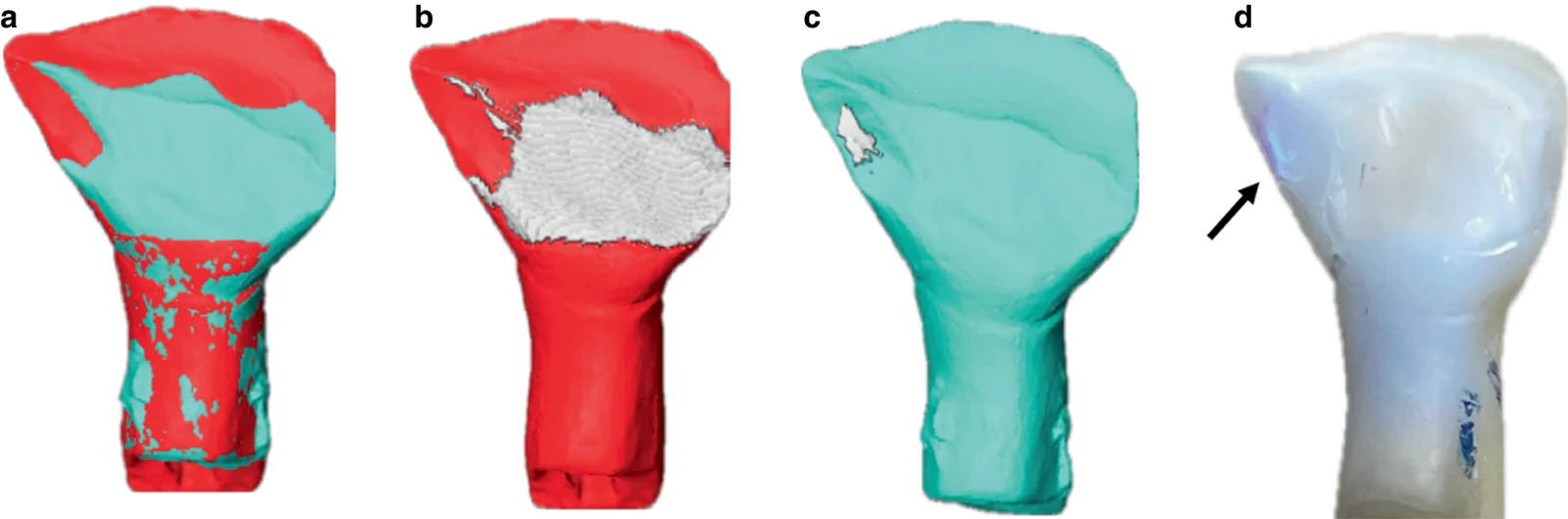
Avizo evaluation procedure. **a** Overlay of intraoral scans using the Avizo software. The T0 scan (tooth before bonding) is shown in turquoise. The T1 scan (tooth after retainer removal) is shown in red. **b** Subtraction of the T1 scan from the T0 scan. The grey area indicates the amount of enamel loss after retainer removal. **c** Subtraction of the T0 scan from the T1 scan. The grey area indicates the amount of composite residue after retainer removal. **d** The same tooth under ultraviolet (UV) light after retainer removal. The black arrow marks the composite residue, which corresponds to residue found in (**c**) Avizo-Auswerteverfahren. **a** Überlagerung von intraoralen Scans mit der Software Avizo. Der T0-Scan (Zahn vor der Verklebung) ist in türkis dargestellt, der T1-Scan (Zahn nach Entfernung des Retainers) ist rot. **b** Subtraktion des T1-Scans vom T0-Scan. Der graue Bereich zeigt das Ausmaß des Schmelzverlustes nach der Entfernung des Retainers an. **c** Subtraktion des T0-Scans vom T1-Scan. Der graue Bereich zeigt die Menge an Kompositresten nach Entfernung des Retainers an. **d** Derselbe Zahn unter ultraviolettem (UV) Licht nach Entfernung des Retainers. Der schwarze Pfeil markiert den Kompositrest, der dem in (**c**) gefundenen Rest entspricht

Following the removal of the retainer, a new 3D surface (Trios 3) and tomography (OCT) scan of each tooth was recorded (T1). Using the Avizo software (Version 8.1.1; ThermoFisherScientific, Darmstadt, Germany), the 3D surface scans were transformed into a volume, and the T0 and T1 scans were aligned by the program. The enamel loss was then quantified by subtracting the T1 volume from the T0 volume and calculating the resulting difference in volume and area. A similar procedure was repeated for the composite and adhesive residue, with the T0 scan being subtracted from the T1 scan (Fig. 2).

From the OCT scan 25 slices (B-scans) per tooth were equidistantly selected and evaluated using a dichotomous scoring system regarding the enamel loss and a four-point scoring system for the composite residue. The absence of composite or adhesive residue was indicated by a grade 0, whereas a grade 1 indicated the presence of adhesive residue alone. A grade 2 indicated the presence of composite residue alone. Finally, a grade 3 was assigned when both adhesive and composite residue were visible.

### Statistical evaluation

The four-point scoring system was then subjected to quantification via the addition of the scores for each tooth, thus, enabling a statistical comparison. To ensure the reliability of the results, intra- and interobserver differences were excluded using a testing system. Ten OCT scans were compared on a different day by the original evaluator and a different evaluator familiar with the evaluation procedure.

In order to assess statistically significant differences between the FIT and non-FIT group, a two-tailed t‑test with a confidence level of 95% and *P*-value of < 0.05 was conducted using the GraphPad Prism software (version 10.4.0, GraphPad, Boston, MA, USA). Differences in removal time, enamel loss and composite and bonding residue were probed.

## Results

### Removal time

**Fig. 3:**
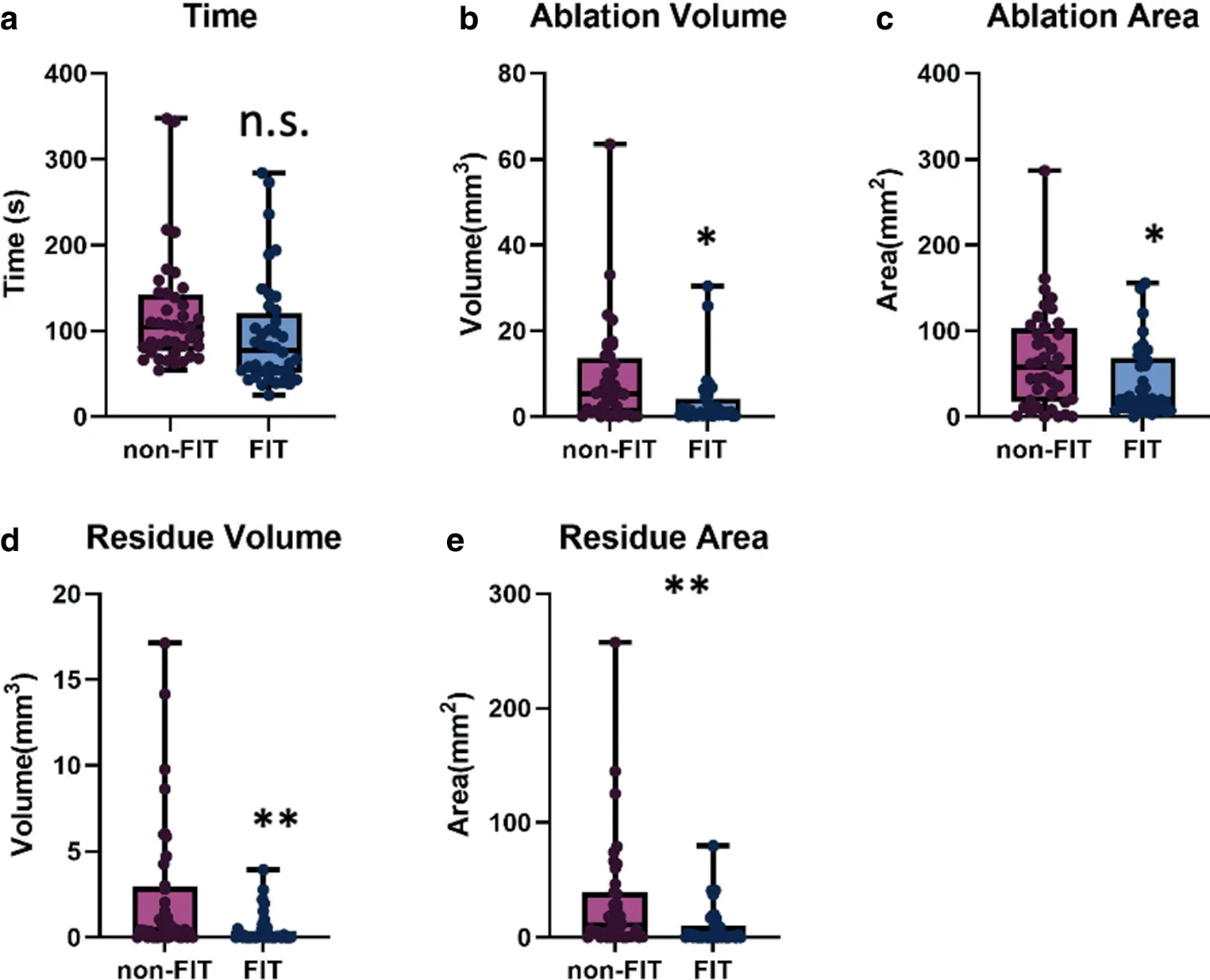
Quantification of time, enamel loss and composite residue. **a** Box-and-whisker plot comparing the time needed for removal using the fluorescence-aided identification technique (FIT) and non-FIT method. No significance (n. s.) is found in relation to the time. Box-and-whisker plots showing enamel ablation in volume (**b**) and area (**c**). Box-and-whisker plots showing composite residue in volume (**d**) and area (**e**). Statistically significant differences regarding the methods are present in all plots (*p < 0.05, **p < 0.01) Quantifizierung von Zeit, Schmelzverlust und Kompositresten. **a** Box-Whisker-Plot zum Vergleich der für die Entfernung benötigten Zeit mit der fluoreszenzgestützten Identifikationstechnik (FIT) und der Nicht-FIT-Methode. Es wurde keine Signifikanz (n.s.) in Bezug auf die Zeit festgestellt. Box-Whisker-Plots, die den Schmelzabtrag in Volumen (**b**) und Fläche (**c**) zeigen. Box-Whisker-Plots, die den Kompositrückstand in Volumen (**d**) und Fläche (**e**) zeigen. Statistisch signifikante Unterschiede zwischen den Methoden sind in allen Diagrammen vorhanden (*p < 0,05, **p < 0,01)

No significant difference in removal time was found between FIT and non-FIT (Fig. 3a). The mean time with UV light was 94.9 s and the mean time with standard white light was 119.3 s, showing a trend towards a faster removal time in the FIT group, but with a *P*-value of 0.097 the t‑test was not significant.

The null hypothesis for removal time had to be accepted.

### Enamel loss

**Fig. 4:**
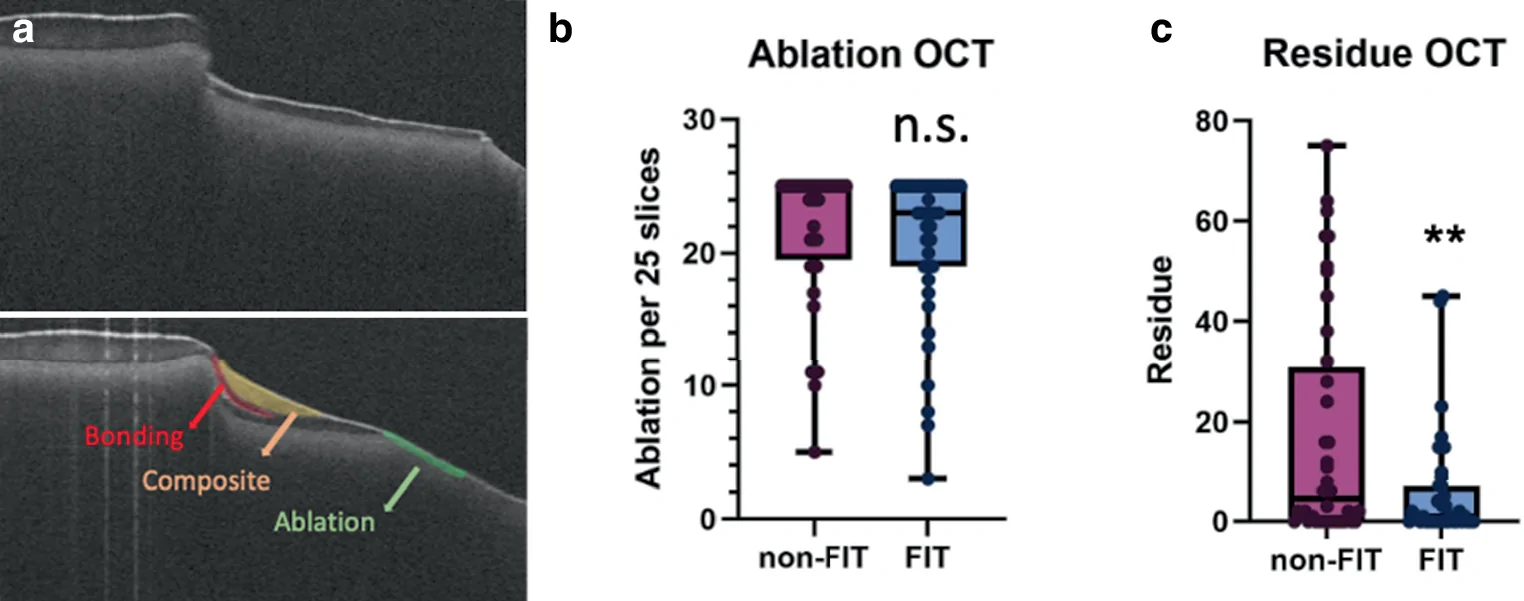
Optical coherence tomography (OCT) evaluation. **a** OCT slices deducted from the same tooth before bonding (top panel) and after retainer removal (lower panel). The ablation is displayed in green, the bonding residue is displayed in red, and the composite residue in orange. Box-and whisker plot showing the comparison of the fluorescence-aided identification technique (FIT) vs. non-FIT group regarding the ablation (**b**) and residue (**c**) analyzed in the OCT slices. The ablation was quantified using a dichotomous score (yes/no) and the summative score of 25 serial slices (**p < 0.01) Auswertung mit optischer Kohärenztomographie (OCT). **a** OCT-Schichten desselben Zahns vor dem Bonding (oberes Feld) und nach der Entfernung des Retainers (unteres Feld). Die Abtragung ist in grün, der Bonding-Rest in rot und der Komposit-Rest in orange dargestellt. Box-Whisker-Plot, der den Vergleich der fluoreszenzgestützten Identifikationstechnik (FIT) mit der Nicht-FIT-Gruppe in Bezug auf die in den OCT-Schichten analysierte Abtragung (**b**) und den Rest (**c**) zeigt. Die Abtragung wurde anhand eines dichotomen Scores (ja/nein) und des summativen Scores von 25 seriellen Schichten quantifiziert (**p < 0,01)

The analysis with Avizo showed that the volume of lost enamel was significantly lower in the FIT group than in the non-FIT group (*p* < 0.05, Fig. 3b). The same was observed for the ablated area (*p* < 0.05, Fig. 3c). Ablation was seen on an average of 20.78 out of the 25 slices of the OCT B‑scans scan of a tooth treated with FIT and 21.73 for the non-FIT group. However, this trend towards FIT was not significant (Fig. 4b).

Thus, while both methods resulted in enamel loss as detected by OCT, FIT reduced the amount of enamel loss and, therefore, the null hypothesis that composite removal with FIT results in the same enamel loss as that with standard white light had to be rejected.

### Composite and adhesive residue

The intraoral surface scans showed that the volume of composite and bonding adhesive residue was significantly lower when FIT was used, compared to when non-FIT was used (*p* < 0.01, Fig. 3d). The same was observed for the area of composite remaining on the teeth (*p* < 0.01, Fig. 3e). Furthermore, this was confirmed by the evaluation of the OCT scans (*p* < 0.01, Fig. 4c). Here, the mean score of 5.88 in the FIT group was significantly lower than the mean score of 16.93 in the non-FIT group.

The null hypothesis that composite removal with FIT results in the same composite and adhesive residue as that with standard white light had to be rejected.

## Discussion

This in vitro study demonstrates that removal of retainers using FIT resulted in less enamel loss and less composite residues, while not accelerating removal time.

Indeed, it was unexpected to find that retainer removal was not faster with FIT, since other studies have shown FIT to be faster for removal of buccal brackets [31, 42], trauma splints [10], and fillings [9, 20]. However, it is important to mention that a previous study on the removal time of lingual brackets showed FIT to be more time consuming [13]. Overall, a possible explanation for these inconsistent results could be that interpersonal time differences during composite removal appear to be more pronounced [32] than the technique itself. Moreover, it can be hypothesized that with a larger number of operators or teeth, the trend towards FIT shown in this study would also be significant for lingual retainer removal, as with a larger sample size, interpersonal differences will play a smaller role and, therefore, have less influence on the result. With a larger sample size, it would also be possible to investigate the influence of experience on the use of FIT, as two studies investigating FIT in relation to the removal of fillings [20] and brackets [42] have shown that FIT is particularly useful for experienced dentists. It, therefore, appears that a larger standard deviation must be used for a future sample size analysis.

Another important finding of this study was that removal of retainers with FIT resulted in less enamel loss. This is in contrast to a previous study showing that the removal of lingual brackets leads to larger enamel defects when FIT was used [13]. However, the operators of that study used a fluorescent composite as well as a florescent bonding adhesive, whereas we used in our study only a fluorescent composite. It is, therefore, possible that the use of a fluorescent bonding adhesive, which has been observed to penetrate the enamel by an average of 8.7 µm [38], could lead to more enamel loss when using FIT. Given the observed difference in morphology between lingual and buccal tooth surface [5], it is also plausible that the effect of FIT in combination with a fluorescent bonding adhesive may be greater on the lingual side. Consequently, the use of a fluorescent bonding adhesive in conjunction with FIT cannot be recommended. Nevertheless, it is important to mention that enamel ablation was visible on a microscopic level in almost every slice in the OCT scans (Fig. 4a). OCT is recognized as a highly reliable tool for analyzing enamel thickness and structure [1, 2, 22, 27, 30], but it should be noted that the OCT evaluation of enamel loss was performed with a dichotomous scoring system, which did not allow absolute quantification of the loss. The morphological analysis of the intraoral scans is, therefore, a more relevant method for the analysis of enamel loss. For future studies, a finer scoring scheme considering the volumetric changes of the residues would be preferable [33].

Finally, it was observed that the removal of the retainer with FIT resulted in a reduced amount of residual composite and adhesive. It is imperative to remove composite due to the fact that residual composite can lead to elevated plaque accumulation rates [4, 36] and, thus, result in an augmented risk of caries or gingivitis/periodontitis. Furthermore, it can jeopardize the stability of the placement of new retainers, as old composites weaken the strength of new composite bonding [3, 34]. The composite and adhesive residue was analyzed with intraoral and OCT scans. The results from both methods were consistent. This further substantiates the finding that FIT leads to less residue on the teeth. A comparison of the composite residue results shown in the overlays of the intraoral scans with the tooth under UV light demonstrated the reliability of using the alignment of intraoral scans as an evaluation technique ([20]; Fig. 2c,d), being supported by the fact that scanning a single tooth, as done in this study, proved to give the most accurate results [12]. Some residue remained even in the FIT group, which could be explained by the inability of the adhesive used in this study to fluoresce. However, an adhesive with light-emitting properties, while ensuring complete removal, could potentially lead to an increase in enamel loss, as previously discussed.

However, the design of this study is not without its limitations. First, the use of bovine teeth is problematic as, although their microscopic structure is very similar to human teeth [35, 37, 46], there is some variance in the size of bovine incisors, which might be one reason for the large statistical variation (Fig. 3). To eliminate any discrepancies, the teeth were sorted equally to both operators. Due to the disparity in size between human and bovine teeth, direct transfer of mean volume and area of enamel loss and composite residue is not possible. A study utilizing human teeth may provide more reliable data. Another limitation is the in vitro approach. Although the clinical setting was reproduced as closely as possible, patient movement and salivation cannot be replicated in an in vitro study. The handheld UV light, despite its efficiency, cost-effectiveness [47] and practicality in a training model, may be inconvenient for daily use; the use of a UV-emitting handpiece would be beneficial. Finally, the time that the retainers remained on the teeth is a limitation. Although the fluorescence properties of the composite do not affect their handling and quality [39], they do decrease by up to 30% over the course of 10 years [21, 44]. However, as the failure of retainers is most likely to occur within the first 3–6 months [18, 24, 41], this should not hinder most removal processes and cannot be regarded as a significant limitation of this study.

A rigorous study design and meticulous experimental setup were employed to ensure reproducibility during the course of the experiments, as well as for potential future replicates. The materials and programs used in this study are widely available in the commercial sector, thereby, ensuring the replicability of the study design. Despite the uniformity in operator instruction, the conductors may introduce variability, as interpersonal differences and experience can influence outcomes. This issue has already previously been the subject of discussion.

## Conclusion

This in vitro study shows that the removal of a fixed lingual orthodontic retainer using a fluorescence-assisted identification technique (FIT) resulted in less enamel loss and less composite and adhesive residue, while not reducing the time required for removal.

## Data Availability

Data will be made available by the corresponding author upon reasonable request.
